# Hydroponics with Microalgae and Cyanobacteria: Emerging Trends and Opportunities in Modern Agriculture

**DOI:** 10.3390/biotech13030027

**Published:** 2024-07-22

**Authors:** Prabhaharan Renganathan, Edgar Omar Rueda Puente, Natalia V. Sukhanova, Lira A. Gaysina

**Affiliations:** 1 Department of Bioecology and Biological Education, M. Akmullah Bashkir State Pedagogical University, 450000 Ufa, Russia; prabhaharan06@gmail.com (P.R.); n_suhanova@mail.ru (N.V.S.); 2 Departamento de Agricultura y Ganadería, Universidad de Sonora, Blvd. Luis Encinas y Rosales, Hermosillo 83000, Sonora, Mexico; edgar.rueda@unison.mx; 3 All-Russian Research Institute of Phytopathology, 143050 Bolshye Vyazemy, Russia

**Keywords:** biostimulant, circular economy, bioremediation, fertilizers, sustainable agriculture, urban farming, climate change resilience

## Abstract

The global population is expected to reach 9.5 billion, which means that crop productivity needs to double to meet the growing population’s food demand. Soil degradation and environmental factors, such as climate events, significantly threaten crop production and global food security. Furthermore, rapid urbanization has led to 55% of the world’s population migrating to cities, and this proportion is expected to increase to 75% by 2050, which presents significant challenges in producing staple foods through conventional hinterland farming. Numerous studies have proposed various sustainable farming techniques to combat the shortage of farmable land and increase food security in urban areas. Soilless farming techniques such as hydroponics have gained worldwide popularity due to their resource efficiency and production of superior-quality fresh products. However, using chemical nutrients in a conventional hydroponic system can have significant environmental impacts, including eutrophication and resource depletion. Incorporating microalgae into hydroponic systems as biostimulants offers a sustainable and ecofriendly approach toward circular bioeconomy strategies. The present review summarizes the plant growth-promoting activity of microalgae as biostimulants and their mechanisms of action. We discuss their effects on plant growth parameters under different applications, emphasizing the significance of integrating microalgae into a closed-loop circular economy model to sustainably meet global food demands.

## 1. Introduction

According to the United Nations Department of Economic and Social Affairs, the global population is anticipated to reach 9.5 billion by 2050, which presents significant challenges for modern agricultural practices [1]. To meet the increasing food demands of the growing population, these current practices must double crop productivity. Over the last few decades, the frequency and intensity of soil degradation and environmental factors have increased owing to real-time climatic events and human activities, posing uncertainty in crop production systems, which could severely threaten global food security [2,3]. Environmental factors include water shortages, high levels of salt, nutrient imbalances (including mineral toxicity and deficiencies), and heat waves, significantly impacting crop productivity worldwide. All of these factors can cause a notable decline in the yield of staple food crops, such as wheat, maize, millet, sorghum, and rice, with yearly decreases ranging from 7% to 23% [4]. Therefore, comprehending and alleviating the impacts of environmental stressors are crucial to ensure agricultural systems’ sustainability and resilience.

Over the last two decades, more than half of the world’s population (i.e., 55%, have migrated to urban areas), which could increase to 75% by 2050. As the population grows and urbanization expands, major cities could face significant challenges in producing staple food crops through conventional hinterland farming [3,5]. To address this issue, cultivators are exploring intensive agricultural production strategies to deliver high crop yields and economic returns despite the limited availability of arable land resources and the high cost of labor [6]. In this regard, several studies have proposed distinct sustainable farming techniques in urban areas to combat the shortage of farmable land and increase food security by improving the resilience of food supply chains and resource efficiency [7]. For instance, soilless farming methods, such as hydroponics, aeroponics, and aquaponics, have recently gained worldwide popularity [8]. In particular, hydroponic systems are the most desirable among these soilless cultivation methods because they reduce waste solution drainage, occupy less growing space, and potentially reduce the utility of mineral fertilizers and production costs [9]. This advanced system can also protect crops against yield loss due to soil-borne diseases and environmental factors. Furthermore, it promotes high-quality fresh products, essential for maintaining a healthy lifestyle and a nutritious diet [10]. In 2021, a survey report on the hydroponics market was published stating that vegetable production in hydroponic systems is expected to grow at a compound annual rate of 20.7% from 2021 to 2028 [11]. To further optimize the efficiency of hydroponic systems, growers can adopt smart farming technology, which employs the Internet of Things (IoTs) to automatically monitor and manage target plants using sensors, microcontrollers, website platforms, and mobile applications [12]. Despite the several benefits of hydroponic systems, some growers have expressed concerns about the high installation costs associated with them [13].

Using chemical fertilizers in a conventional hydroponic system can significantly impact the environment and lead to eutrophication and resource depletion. Hence, it is critical to reassess the conventional hydroponic system and develop sustainable alternatives [14,15]. Deploying biostimulants has garnered significant interest, as they offer a sustainable and environmentally friendly approach to crop management. In particular, biostimulants are specially formulated to enhance plant growth and development by improving nutrient uptake, stress tolerance, and other physiological functions. Commercially available biostimulant products mainly comprise naturally occurring organic substances such as plant extracts, amino acids, and microbial metabolites [16,17]. As per the European Biostimulant Industry Council, biostimulants are categorized as organic materials that promote plant nutrition regardless of their nutrient composition. These products are designed to enhance one or more characteristics of a plant or the surrounding soil (i.e., rhizosphere), including nutrient utilization, stress tolerance, quality traits, or access to limited soil nutrients [18]. Among the various categories of biostimulants, seaweed extracts have been studied extensively and represent an essential category in microbial inoculants compared to microalgae [19]. However, microalgae and cyanobacteria have emerged as promising renewable bioactive resources in agriculture for the development of plant biostimulants [20].

Microalgae represent a diverse group of photosynthetic phytoplanktonic organisms comprising cyanobacteria and eukaryotic species, such as green algae, euglenoids, and diatoms, which can thrive in both freshwater and marine environments [21]. Studies have reported the biostimulant properties of microalgae biomass for plant growth promotion and soil fertility [22,23,24,25], which are ascribed to the synthesis of biologically active molecules, including phytohormones, amino acids, phenolics, osmolytes, and sulfated polysaccharides [26]. All of these metabolites have been determined to enhance crop performance, impart resistance against abiotic stresses, induce plant defense response against pathogens and infections, and improve essential nutrient uptake, including nitrogen (N), phosphorus (P), potassium (K), and other minerals [27,28,29]. In previous studies, distinct classes of microalgae metabolites have been detected, with plant-stimulating properties [25]; however, their modes of action and the effects on plant physiology are not clearly understood [26]. Furthermore, the impact of microalgae-derived biostimulants and their application in hydroponic systems have rarely been discussed in the literature. Therefore, there is an urgent need to investigate the underlying mechanisms of microalgae-derived metabolites and their application methods in hydroponic systems to meet the growing demand for sustainable agricultural practices in urban areas.

This review summarizes the mechanism of action of various microalgae metabolites and their effects on plant growth performance in hydroponic systems. Although there is limited literature on this topic, existing studies have not explored the functionalities of microalgae metabolites in plant growth, especially in hydroponic systems. Our review highlights the significance of integrating hydroponics and microalgae in a closed-loop circular bioeconomy model. The primary objective of this review is to promote the commercialization of microalgae-derived metabolites for modern agriculture and emphasize their efficacy and sustainability in meeting global food demands.

## 2. Microalgae as Biostimulants

Biostimulants encompass a diverse range of organic substances or beneficial microorganisms, including bacteria, fungi, and algae, which are directly applied to plants at various growth stages to regulate plant physiological and developmental functions [17,30,31,32,33]. Growing evidence suggests that both photosynthetic prokaryotic cyanobacteria and eukaryotic microalgae are highly efficient in promoting plant growth and have significant potential for developing biostimulants [34]. Studies conducted at laboratory and greenhouse scales have demonstrated the significant effects of applying live cell suspensions, dry biomass, cell extracts, or hydrolysates of microalgae on the growth of economically important cereals, such as wheat, corn, rice, and sorghum, as well as certain spice crops and vegetables (Table 1). These studies indicate consistent improvements in plant growth and performance followed by soil inoculations or extract applications, although the specific plant responses may vary depending on the microalgal strain, method of application, and experimental conditions [22,34,35].

Microalgae synthesize a wide range of biostimulatory components, such as phytohormones, polysaccharides, terpenoids, and phenols, which effectively promote plant nutrient absorption, induce stress resistance, improve crop quality, optimize soil–water efficiency, reinforce root structure, and support vital physiological plant functions, including respiration, photosynthesis, iron absorption, and nucleic acid synthesis [22,51,52]. Several microalgal species have been identified as beneficial microorganisms and are widely used for various industrial and commercial purposes, including the genera *Haematoccocus* sp., *Chlorella* sp., *Dunaliella* sp., *Isochrysis* sp., *Porphyridium* sp., *Nannochloropsis* sp., and *Spirulina* sp. [53]. However, metabolites derived from a few microalgal species of *Dunaliella* sp., *Chlorella* sp., *L. platensis*, *Acutodesmus* sp., *C. elenkini*, and *Scenedesmus* sp. are used as plant growth promotors in agriculture [29,51].

### 2.1. Phytohormones

**Figure 1:**
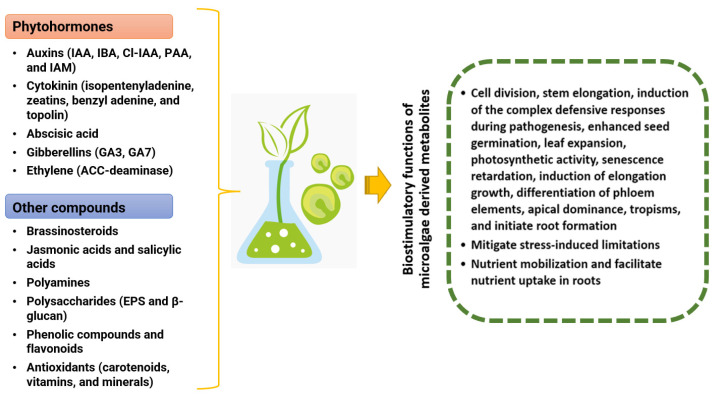
Infographic showing the variety of biostimulant compounds produced by microalgae and their effect on plant growth.

Phytohormones serve as chemical messengers that regulate plant physiological and developmental functions. These plant-derived small signaling molecules are produced from various essential metabolic pathways in low concentrations [54,55]. Auxins, cytokinins, gibberellic acid (GA 3), abscisic acid (ABA), and ethylene are well-known phytohormones found in microalgae [56]. Microalgae accumulate phytohormones in their cells and release them into the extracellular environment, which has similar regulatory functions to those of terrestrial plants [57]. Microalgae phytohormones could potentially serve as regulatory agents in microalgae’s growth, development, and metabolism (Table 2). These microalgal phytohormones could also regulate many critical physiological plant functions, including cell division, growth and differentiation, organogenesis, seed germination, dormancy, senescence, and response to biotic and abiotic stresses [55,58] (Figure 1).

#### 2.1.1. Auxins

**Figure 2:**
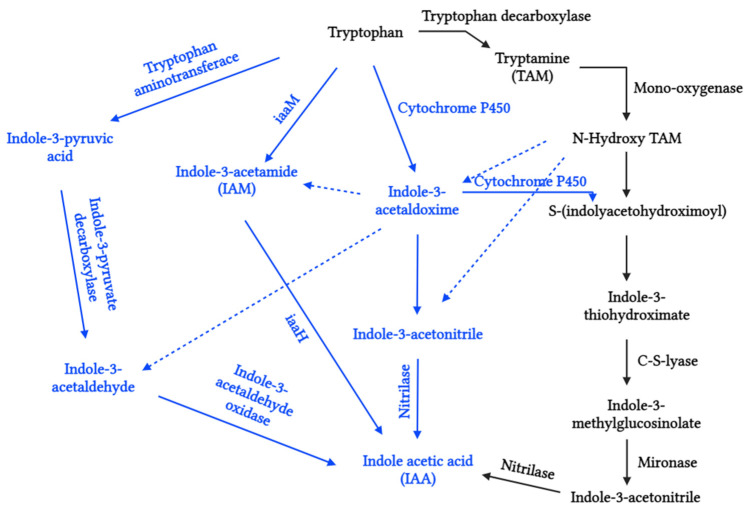
Tryptophan-dependent pathway of IAA biosynthesis. Solid arrows represent the enzymes or genes responsible for the identified steps. Dashed arrows indicate the proposed pathway, although it remains undetermined. The black-colored pathway is identified in algae species, whereas the blue-colored pathway is found in higher plants.

Auxins are a group of phytohormones that have been the subject of extensive research. They consist primarily of indole-3-acetic acid (IAA), indole-3-butyric acid (IBA), 4-chloroindole-3-acetic acid (ClIAA), and 2-phenylacetic acid (PAA) [56,91]. The biosynthesis of auxins occurs through the indole-3-pyruvic acid (IPA) pathway, indole-3-acetamide (IAM) pathway, indole-3-acetaldoxime (IAOx) pathway, and tryptamine (TAM) pathway, of which TAM and IPA are the most common pathways found in leaf primordia, young leaves, and fruits (Figure 2). Perhaps, the most probable pathway for auxin biosynthesis in algae is the TAM pathway. The presence of the tryptophan decarboxylase enzyme has been demonstrated in the microalga *Chlamydomonas reinhardtii*. Several auxin biosynthetic enzymes documented in higher plants, such as C-S lyase and nitrilases, have also been observed in *Ectocarpus siliculosus*, *Ostreococcus lucimarinus*, *Micromonas pusilla*, *Chlorella variabilis*, *Volvox carteri*, etc. [92]. Additionally, cyanobacterial genera, such as *Nostoc* sp., *Chlorogloeopsis* sp., *Calothrix* sp., *Plectonema* sp., *Gloeothece* sp., *Anabaena* sp., *Cylindrospermum* sp., and *Anabaenopsis* sp. [93], and microalgae genera, such as *Chlorella* sp., *Coenochloris* sp., *Acutodesmus* sp., and *Scenedesmus* sp., are the primary sources of auxin biosynthesis.

Auxins play a crucial role in regulating various physiological processes such as cell elongation (via activation of the plasmalemma H^+^-ATPase), differentiation of phloem, apical dominance, tropisms, initiation of root formation, and abiotic stress tolerance [56,94,95,96,97,98]. Endogenous auxins, such as IAA and indole-3-acetamide (IAM), were present in significant concentrations, ranging from 0.50 to 71.49 nmol IAA g^−1^ DW and 0.18 to 99.83 nmol IAM g^−1^ DW, respectively, in 24 microalgal biomass extracts of *Chlorophyceae* sp., *Trebouxiophyceae* sp., *Ulvophyceae* sp., and *Charophyceae* sp. However, IAA predominated over IAM in 19 microalgal species [99]. When *Scenedesmus* sp. extracts containing 5.96 µg g^−1^ of IAA were applied to Petunia plants, it resulted in a significant increase in root dry weight (49%), flower dry weight (20%), and flower fresh weight (22%) [100].

Cyanobacteria-produced auxin (0.20 to 1.63 µg mL^−1^ IAA) significantly impacted plant vegetative growth in a hydroponic system. Interestingly, the cyanobacteria produced more endogenous auxin than exogenous auxin in the presence of plants. This could be attributed to the plants releasing specific signals to trigger the production of higher levels of auxins in the cyanobacteria [101]. The IAA-producing microalgae *C. vulgaris* resulted in high leaf numbers, long shoots, and significant root initiation without branching and leaf expansion. Similarly, *Anabaena oryzae* and *N. muscorum* stimulated a 1.5-fold increase in the fresh weight of soybean callus [102]. Furthermore, the auxin derived from the mangrove-root-associated cyanobacterium *Phormidium* sp. enhanced seed germination by 40% and induced multiple roots in tobacco callus under salinity conditions. Similarly, Karthikeyan et al. [103] confirmed that auxins in *Calothrix ghosei*, *Hapalosiphon intricatus*, and *Nostoc* sp. stimulated wheat seeds’ germination percentage, radicle, and coleoptile length.

#### 2.1.2. Cytokinins

**Figure 3:**
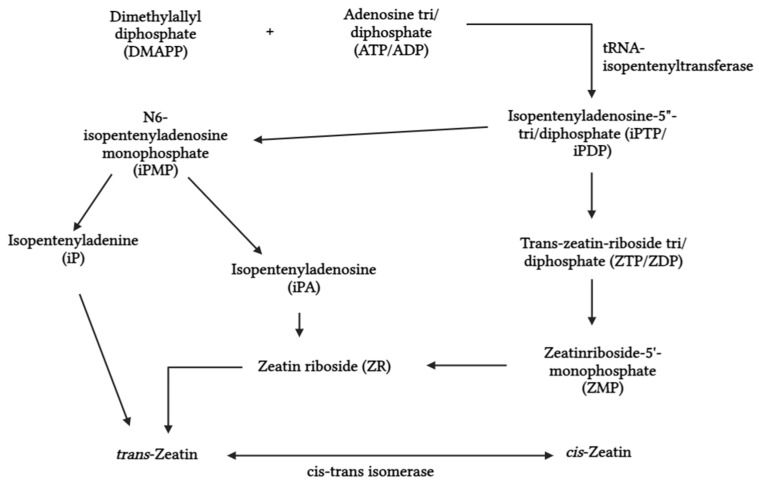
Biosynthesis of cytokinin.

Cytokinins are a class of phytohormones that contain N^6^-substituted adenine-based molecules with either aromatic or isoprenoid side chains. Isoprenoid cytokinin is synthesized through two distinct pathways. The direct pathway involves the conversion of AMP and pyrophosphate into N6-isopentenyladenosine monophosphate (iPMP), which can be regulated by the enzyme isopentenyltransferase (IPT) (Figure 3). The indirect pathway involves the synthesis of isoprenoid cytokinin by structural modifications of tRNAs that contain cis-zeatin [87]. These compounds are prevalent in microalgae, such as *Protococcus* sp., *Chlorella* sp., and *Scenedesmus* sp., which contain predominant cytokinins like isopentenyladenine, zeatins, benzyl adenine, and topolin [91,104]. Cytokinins are crucial in promoting plant cell division, enlargement, and differentiation, as well as other developmental functions like chloroplast and vascular tissue development, root and shoot meristem function, apical dominance, and leaf senescence [55,105]. They also facilitate root nodule formation and enhance plant–microbe interaction [106].

Cytokinin-deficient plants tend to have smaller apical meristems and stunted shoots [96]. A recent study revealed that *Stigeoclonium nanum*, a cytokinin-derived microalga, contains cis-zeatin and isopentenyladenine (21.40 nmol g^−1^ DW), which were found to be the most predominant compounds, followed by trans-zeatin and dihydrozeatin in low levels, in addition to free bases and their ribosides [99]. Moreover, benzyl adenine, found in the algal extract of *A. oryzae* and *N. muscorum*, significantly enhanced shoot length and leaf number, leading also to high root initiation, in tomatoes [102]. Cytokinins derived from *Desmodesmus subspicatus* extracts grant a 30% increase in plant biomass, enhanced cell division, leaf chlorophyll contents, enhanced fresh weight, and increased cotyledon size in cucumbers [107]. Furthermore, cytokinins have been found to improve abiotic tolerance in target crops. For instance, *Nannochloropsis* sp. alleviated water and N stress in tomato plants [108].

#### 2.1.3. Gibberellic Acid

Gibberellic acid (GA), a diterpene phytohormone, is extensively involved in almost all growth phases of higher plants and microalgae [109]. Moreover, GA plays a crucial role in the abiotic stress tolerance of plants; for instance, GA3, the most bioactive form of GA, ameliorates soil salinity tolerance in *Z. mays* by enhancing the membrane permeability and plant nutrient uptake, which results in better seedling growth and establishment under toxic conditions [110]. In almost all 24 strains of microalgae, 18 to 20 endogenous GAs were detected in 4 days of growth, with concentrations ranging between 342.7 pg mg^−1^ DW and 4746.1 pg mg^−1^ DW in *Raphidocelis subcapitata* and *Coelastrella terrestris*, respectively. However, slower-growing strains of microalgae (*C. terrestris*, *Gyoerffyana humicola*, *Nautococcus mamillatus*, and *Chlorococcum ellipsoideum*) accumulated higher amounts of intracellular GAs than the fast-growing strains (*R. subcapitata* and *Coelastrum excentrica*) [111].

The aqueous extract of *Parachlorella kessleri* is rich in auxin and GAs, which significantly improved seed germination and early seedling growth parameters, leaf elongation, chlorophyll pigment, and accumulation of sodium and P in roots and shoots of *Vicia faba* [112]. Furthermore, extracellular extracts of *Scytonema hofmanni* can produce GA3, which alleviates the adverse effects of homeostasis caused by salinity stress in *O. sativa* [113]. Similarly, *C. vulgaris* extracts containing GA3 reduced the adverse effects of heavy metal stress and imparted a defense mechanism against lead and cadmium [114]. GA3 has many commercial applications in farming industries to improve seed germination and growth rates, prevent leaf senescence, increase fruit size, and delay fruit ripening [115].

**Figure 4:**
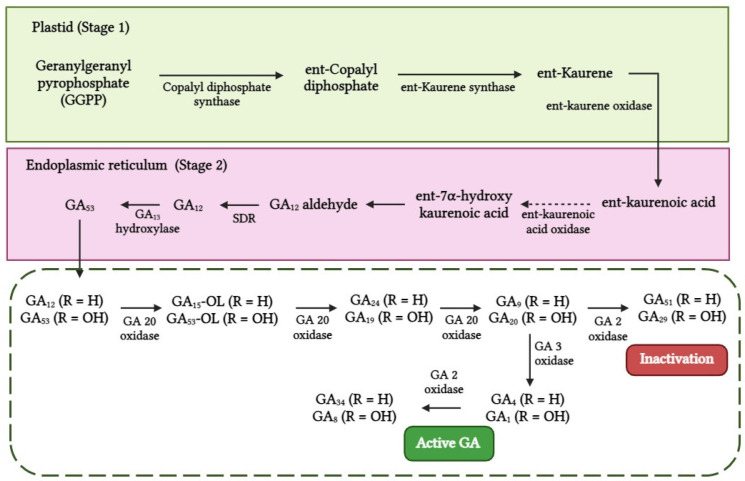
Biosynthesis of gibberellic acid.

Gibberellin is synthesized using either the mevalonic acid or methylerythritol phosphate pathway. GA synthesis in higher plants is primarily carried out via the methylerythritol phosphate pathway, which occurs in plastids. The primary process of GA biosynthesis involves the cyclization of geranylgeranyl pyrophosphate (GGPP) into copalyl pyrophosphate, followed by its further conversion into ent-kaurene (Figure 4), with the action of copalyl pyrophosphate synthase (CPS) and ent-kaurene synthase, respectively. Enzymes such as copalyl pyrophosphate synthase, ent-kaurene synthase, and ent-kaurenoic acid oxidase have not been documented in algae. However, in the green alga *C. reinhardtii*, the enzyme GA-20 oxidase has been identified. This enzyme is closely related to the late-stage enzymes involved in GA synthesis in *A. thaliana*. This suggests that the GA synthesis mechanism in algae could be similar to that of higher plants [92].

#### 2.1.4. Ethylene

**Figure 5:**
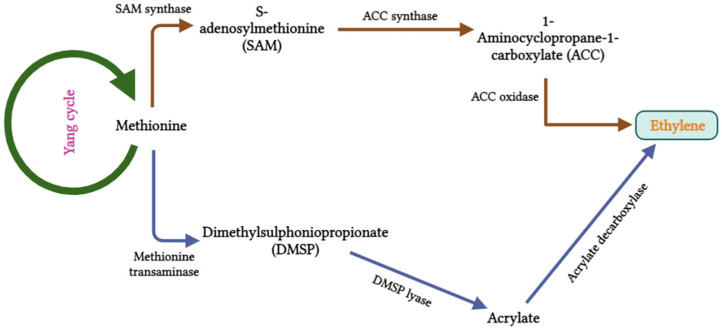
Biosynthesis of ethylene.

Ethylene is a well-known unsaturated two-carbon molecule vital in fruit ripening, organ abscission, and several other plant developmental processes. It regulates seed germination, flowering, leaf senescence, sex determination, and response to various stress factors [116,117]. In the ethylene biosynthesis pathway, methionine serves as a precursor for the production of dimethylsulphoniopropionate (DMSP) (dimethyl-β-propiothetin). The DMSP lyase enzyme converts DMSP into acrylate and dimethyl sulfide. Acrylate is ultimately transformed into ethylene by acrylate decarboxylase (Figure 5). In contrast, a different pathway for ethylene biosynthesis has been reported in Haematococcus pluvialis, which is similar to that in higher plants. In H. pluvialis, ethylene biosynthesis is initiated by L-methionine, which is converted to S-adenosylmethionine (SAM/AdoMet), 1-aminocyclopropane-1-carboxylic acid (ACC), and finally to ethylene through the enzyme ACC oxidase. In H. pluvialis, ACC oxidase is activated by Co^2+^, Mn^2+^, and Ag^2+^ and inhibited by Cu^2+^ and salicylhydroxamic acid. On the other hand, in plants, this enzyme is activated by Fe^2+^, Mn^2+^, or Cu^2+^ and inhibited by Co^2+^ [92].

Ethylene synthesis has been detected in numerous microalgal species, including *Chlamydomonas* sp., *Chlorella* sp., and *Scenedesmus* sp., and cyanobacteria, such as *Synechococcus* sp., *Anabaena* sp., *Nostoc* sp., *Calothrix* sp., *Scytonema* sp., and *Cylindrospermum* sp., being the most active producers [57,118]. Recent studies indicate that *Scenedesmus* sp. and *Arthrospira* sp., have the highest ethylene concentration, estimated between 341 ng g^−1^ and 546 ng g^−1^, respectively [100]. The construction of stable recombinant ethylene-producing cyanobacteria *Synechococcus* sp., has been achieved, representing a significant breakthrough [119]. However, several factors influence the high production of ethylene, including the expression of two different *efe* genes in *Synechocystis* sp., leading to ethylene production rates of approximately 200 nL mL culture^−1^ h^−1^ OD_730/750_^−1^. Incorporating *efe* genes into either a self-replicating plasmid [120] or the chromosome [121] led to the recording of a high ethylene productivity of 171 mg L culture^−1^ d^−1^ using dense cultures. Optimizing the *efe* gene’s expression through the modulation of the ribosome binding site of the expression significantly increased ethylene production [122].

#### 2.1.5. Abscisic Acid

Abscisic acid (ABA) is a sesquiterpenoid hormone that plays a crucial role in regulating various developmental processes and stress responses in synthesizing proteins and compatible osmolytes, which enable plants to tolerate biotic and abiotic stresses [123]. However, studies have shown that ABA suppresses shoot growth, leading to a consequential rise in the root-to-shoot ratio [124]. Foliar application of ABA on cucumber and tomato seedlings significantly reduced the transpiration rates and shoot elongations during storage periods, helping to maintain optimal seedling quality and size for transplanting [124,125].

**Figure 6:**
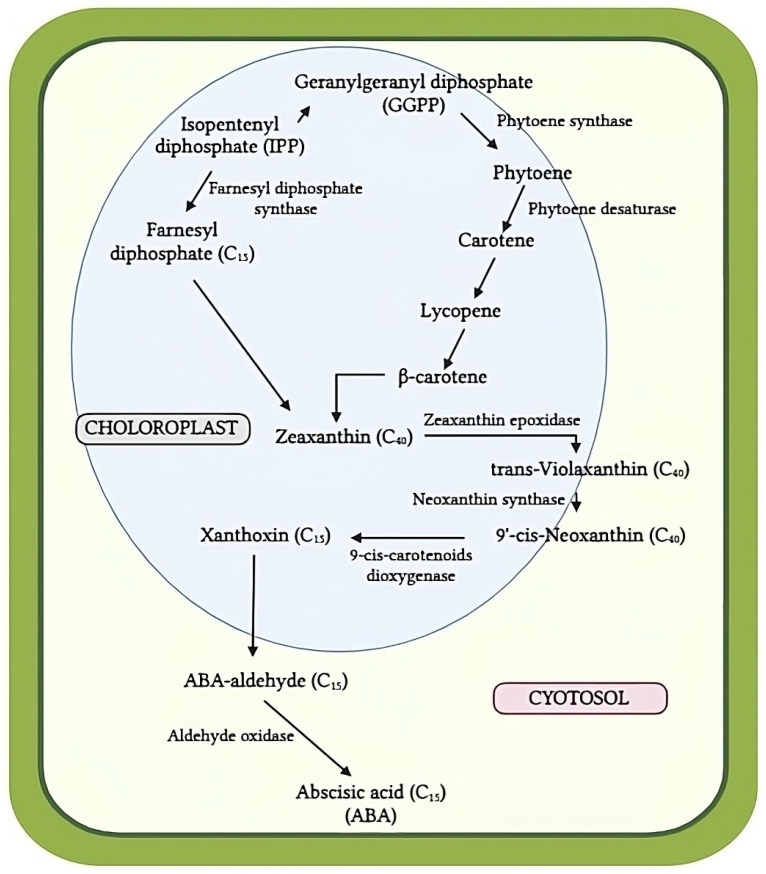
Biosynthesis of abscisic acid. Modified from [92].

ABA biosynthesis occurs either via the precursor isopentenyl pyrophosphate or directly through the breakdown of carotenoids (Figure 6). The first step in ABA biosynthesis is carotenoid synthesis, during which isoprenoids and carotenoids are generated from isopentenyl pyrophosphate (IPP). In the process of carotenoid synthesis, geranylgeranyl pyrophosphate (GGPP) is produced from isopentenyl diphosphate (IPP). Following this, GGPP is converted into phytoene by phytoene synthase (PSY). Phytoene desaturase (PDS) is an enzyme that transforms phytoene into ζ-carotene and then converts it into lycopene, β-carotene, and, finally, zeaxanthin. Alternatively, zeaxanthin may be formed directly from isopentenyl pyrophosphate (IPP) via farnesyl-diphosphate. The first important step in ABA biosynthesis is the conversion of zeaxanthin into trans-violaxanthin by a two-phase de-epoxidation process facilitated by zeaxanthin epoxidase (ZEP). Neoxanthin synthase enzymatically converts trans-violaxanthin into 9-cis-neoxanthin. Subsequently, xanthoxin is produced by the oxidative reduction in 9-cis-violaxanthin and/or 9-cis-neoxanthin, which is catalyzed by the enzyme 9-cis-epoxycarotenoid dioxygenase (NCED). Finally, there are three possible pathways for the last stage of ABA biosynthesis, from xanthoxin to ABA formation, such as ABA aldehyde (as shown in Figure 6), xanthoxinic acid, or abscisic alcohol, which can be intermediate compounds formed before ABA synthesis [92].

Several microalgae groups, such as *C. vulgaris*, *H. pluvialis*, *D. salina*, *C. reinhardtii*, *Cyanidioschyzon merolae*, *S. quadricauda*, *N. oceanica*, and *C. sorokiniana*, can produce high levels of ABA [97,126]. In a study conducted by Plaza et al. [100], the microalga *Scenedesmus* sp. was found to contain high concentrations of auxins (IAA, 5965.0 ng g^−1^), cytokinins (isopentenyl adenine, 45,561.97 ng g^−1^), gibberellins (GA1, 208.81 ng g^−1^), and other hormones, such as abscisic acid (3718.25 ng g^−1^), salicylic acid (156,713.72 ng g^−1^), and jasmonic acid (75.13 ng g^−1^), compared to the bacteria *Arthrospira* sp.

Foliar application of *Scenedesmus* sp. extracts has been found to promote the growth of flowers, shoots, and leaves. Furthermore, the presence of ABA in *Scenedesmus* sp. extracts has been shown to promote proportional root growth. High levels of abscisic acid can inhibit ethylene synthesis, thereby reducing auxin transport and biosynthesis in the root tip. This, in turn, removes the primary root growth inhibitor and promotes root growth [127].

#### 2.1.6. Jasmonic and Salicylic Acids

Jasmonic and salicylic acids are two crucial signaling molecules in plants that play pivotal roles in defense mechanisms against biotic stressors [56]. These molecules are present in a wide range of algae, including *D. tertiolecta*, *D. salina*, *Chlorella* sp., and *Euglena gracilis*, and cyanobacteria *Spirulina* sp. [56]. Salicylic acid is primarily responsible for activating defense mechanisms effective against biotrophic and hemibiotrophic pathogens, whereas jasmonic acid triggers defense against necrotrophic pathogens [128]. Several studies have shown that the signaling pathways mediated by jasmonic and salicylic acids interact with each other to coordinate plant immune responses against pathogens. However, it is noteworthy that pathogens often target these pathways to enhance their virulence and infectivity [129]. Plaza et al. [100] demonstrated the presence of jasmonic and salicylic acids in microalgae *Scenedesmus* sp., with a concentration of 75.13 ng g^−1^ and 15,6714 ng g^−1^, respectively.

### 2.2. Hormone-like Compounds as Biostimulants

In addition to phytohormones, microalgae and cyanobacteria have been found to accumulate low-molecular-weight signaling molecules, including brassinosteroids, polyamines, jasmonic acid, and salicylic acid. These molecules play a crucial role in the regulation of plant growth and development and impart resilience to biotic and abiotic stresses. The ability of microalgae and cyanobacteria to synthesize and accumulate these signaling molecules has made them an attractive target for research in the fields of biotechnology and agricultural sciences. Further studies on the biosynthesis and regulation of these molecules may provide new insights into the mechanisms underlying plant–microbe interactions and lead to the development of novel strategies for crop improvement.

#### 2.2.1. Brassinosteroids

Brassinosteroids are polyhydroxylated steroid compounds that play a crucial role in various physiological and molecular plant processes, such as root and shoot elongation, germination, flowering, vascular differentiation, and fertility. Brassinosteroids also aid in plant response to different biotic and abiotic stressors [130]. Studies have shown that foliar application of brassinosteroids on rice [131], tomatoes [132], and snap beans [133] can help mitigate the adverse effects of heat stress and enhance overall growth performance by increasing the carboxylation efficiency and antioxidant activity in leaves. Bajguz [134] identified seven brassinosteroids compounds in the wild species of *C. vulgaris*, such as typhasterol (0.39 ng g^−1^), teasterone (0.26 ng g g^−1^), 6-deoxoteasterone (0.22 ng g g^−1^), 6-deoxotyphasterol (0.18 ng g g^−1^), 6-deoxocastasterone (0.32 ng g g^−1^), castasterone (0.47 ng g^−1^), and brassinolide (0.07 ng g^−1^). In another study, Stirk et al. [111] identified brassinolide and castasterone as the two most common types of brassinosteroids in 24 microalgal strains. The concentration of brassinosteroids ranged from 117.3 pg mg^−1^ DW in *R. subcapitata* to 977.8 pg mg^−1^ DW in *Klebsormidium flaccidum*.

#### 2.2.2. Polyamines

Polyamines are a class of low-molecular-weight polycations that contain two or more amino groups. They play crucial roles in various physiological functions of plants, including plant growth and development, molecular signaling, cell division, differentiation, totipotency, and biotic and abiotic stress responses [135,136,137]. The most common polyamines in all living organisms are putrescine, spermidine, and spermine [137]. In addition to these compounds, microalgae contain nonspermidine, nonspermine, diaminopropane, and cadaverine [138]. Interestingly, the cell walls of the microalgae *Scenedesmus* sp. and *Chlorella* sp. contain similar conjugated polyamines, such as putrescine, spermidine, and spermine, as found in higher plants [139]. Furthermore, polyamines extracted from cyanobacterium *L. platensis* have been found to promote the growth of lettuce seedlings and exhibit biostimulant effects [140].

#### 2.2.3. Polysaccharides

Polysaccharides are a prevalent type of biopolymer composed of monosaccharides linked through α- or β-glycosidic units [141]. Microalgae cells are known to produce extracellular polymeric substances (EPS), which form a slimy coating that serves as a protective layer around the cell to withstand extreme environmental stressors [142]. The EPS found in different microalgae species, such as *C. reinhardtii*, *Botryococcus braunii*, *D. tertiolecta*, *Porphyridium purpureum*, *Spirulina* sp., *I. galbana*, and *D. salina*, have been extensively studied to gain valuable insights into their chemical compositions, structural properties, biosynthesis mechanisms, and functional characteristics [23].

Polysaccharides constitute up to 46% of the dry weight of microalgal extracts, particularly in *Chlorella* sp., *Chlamydomonas* sp., *Dunaliella* sp., and *Spirulina* sp. [143]. The application of *L. platensis* extracts containing polysaccharides via foliar spraying has been shown to significantly increase plant growth by 20% and 30%, root weight by 230% and 67%, and node size and number by 57–100% and 33–50%, respectively, in tomato and pepper [58]. Similarly, lower concentrations (1 mg mL^−1^) of raw polysaccharides derived from *L. platensis*, *D. salina*, and *Porphorydium* sp. have been found to significantly impact the number of nodes, shoot dry weight, and shoot length of tomato by 75%, 46.6%, and 25.26%, respectively [24]. Moreover, *L. platensis* has been shown to improve carotenoid content and NAD-glutamate dehydrogenase. At the same time, *Porphorydium* sp. has been found to increase chlorophyll a and b, as well as nitrate reductase activities.

#### 2.2.4. Phenolic Compounds

Phenolic compounds are synthesized from amino acids, particularly phenylamine, which can enhance the defense mechanism of plants [144]. In a recent study, *Spirulina* sp. [145] and *Nannochloropsis* sp. [146] were used in the extraction of both essential, including isoleucine, leucine, lysine, methionine, phenylalanine, threonine, tryptophan, and valine, and nonessential amino acids, such as alanine, arginine, aspartic acid, cystine, glutamic acid, glycine, histidine, proline, serine, and tyrosine. The microalga *Scenedesmus* sp. ME02 has been found to contain antioxidant capacity, total phenolics, flavonoids, and carotenoid contents. The antioxidant capacity was evaluated using 2,2-diphenyl-1-picrylhydrazyl and ferric-reducing antioxidant power assays, with values of 3.71 ± 0.11 μmol Trolox eq. g^−1^ DW and 47.01 ± 3.14 μmol Trolox eq. g^−1^ DW, respectively. The total phenolic, flavonoid, and carotenoid contents were measured at 5.40 ± 0.28 mg gallic acid eq. g^−1^ DW, 1.61 ± 0.76 mg quercetin eq. g^−1^ DW, and 0.61 ± 0.05 mg g^−1^, respectively [147]. In an aqueous extract of microalgae *D. salina*, the highest total phenolics content was 8.78 ± 1.49 mg GAE g^−1^ DW, whereas it was 1.30 ± 0.37 mg GAE g^−1^ DW in the methanolic extract. In contrast, the highest phenolic content in a methanolic extract was reported for *Mychonastes homosphaera* at 9.04 ± 0.68 mg GAE g^−1^ DW, whereas in its aqueous extract, it was only 3.00 ± 0.30 mg GAE g^−1^ DW [148]. When compared to *Nannochloris* sp., *Tetraselmis suecica*, and *Microchloropsis gaditana*, which have lower antioxidant activities, ranging from 51.1% to 56.8%, *Phaedactylum tricornutum* had the highest carotenoid (fucoxanthin) and phenolic contents (protocatechuic acid), along with a 65.5% antioxidant activity [149].

## 3. Abiotic Stress Tolerance

The use of microalgae and their byproducts as biostimulants, particularly to ameliorate abiotic stress, has experienced an upward trend in recent years (Table 3). Among the diverse array of metabolites, exopolysaccharides (EPS) derived from both cyanobacteria and microalgae have shown plant immunostimulatory properties and enhance plant growth-promoting activities in the rhizosphere, including mineral complexation, water retention, etc. [26,143]. The application of sulfated polysaccharide extracts obtained from *D. salina* has been found to increase the salinity tolerance in tomato and pepper plants by enhancing the activities of antioxidant enzymes, such as superoxide dismutase (SOD), peroxidase (POD), catalase (CAT), and ascorbate peroxidase (APX) [23]. Similarly, polysaccharide extracts derived from *L. platensis*, *D. salina*, *Porphyridium* sp., and *P. tricornutum* exhibited biostimulatory effects when applied to tomato plants, leading to increased phenylalanine ammonia-lyase and chitinase enzyme activities, higher polyphenol content, improved ROS scavenging, and enhanced biosynthesis of very long-chain fatty acids that make up the leaf cuticular wax [24].

## 4. Modern Agriculture: Hi-Tech Indoor Farming

Traditional agriculture has relied on soil-based farming practices for many centuries. However, because of the negative impact on the environment, the agricultural industry has gradually shifted toward soilless hi-tech indoor farming techniques, i.e., hydroponics, aeroponics, and aquaponics [3]. Soil-based farming practices, particularly monocropping, excessive mineral fertilization, and agricultural land expansion, can lead to biodiversity loss, soil quality deterioration, water pollution, and greenhouse gas emissions [150]. A significant proportion of the greenhouse gas emissions caused by unsustainable geoponic practices is attributable to the unstable, unequal, and unsustainable global food system [151]. Soilless farming techniques have emerged as a potential solution to address the issue of global food insecurity, especially in urban areas [152,153,154]. The hi-tech indoor farming system is designed to grow plants under a fully controlled growth environment that enables growers to produce continuous crops in shorter growing periods, with higher crop productivity, regardless of the climate, soil quality, or availability of cultivable land [155]. Additionally, this advanced technology requires minimal space to grow plants, reduces nutrition consumption, and eliminates the usage of harmful herbicides and pesticides. The global market for indoor farming systems is expected to grow by 18.8% from 2017 to 2023, corresponding to a market size of USD 490.50 million by 2023 [156]. Despite the higher production costs, consumers are willing to pay a premium for indoor farming products owing to their outstanding quality and alignment with the Sustainable Development Goals [154].

### 4.1. Hydroponics

Hydroponics is a method of cultivating plants that does not rely on soil but rather on indoor nutrient-enriched liquid mediums, with or without mechanical support, such as sand or gravel [157]. Hydroponics has gained popularity in the industrial agriculture sector in recent decades due to its numerous advantages over conventional cultivation methods. Furthermore, it has been proven suitable for growing food in outer space; NASA researchers have successfully grown various vegetables, including onions, lettuce, and radishes, using hydroponics in space [153]. Researchers have taken this method to an advanced level to make it more productive, reliable, and water-efficient. The primary benefit of hydroponics is that it eliminates soil-related pests, such as insects, fungi, and bacteria. It is also less labor-intensive, as it does not require weeding, tilling, kneeling, and soil removal. Additionally, hydroponics provides an easier way to control nutrient levels, oxygen supply, and pH balance, leading to more consistent production and better yields [153,158,159].

Hydroponics has experienced a surge in both vegetable and crop production, as yields of fresh produce grown using hydroponics have better quality crops, flavors, and nutritional value than traditional growing methods. Numerous studies have confirmed that hydroponic systems are highly efficient in cultivating green leafy vegetables, cereals, fruits, condiments, fodders, and medicinal plants (Table 4) [13,160,161,162]. In particular, lettuce is the most studied crop grown using hydroponic systems.

#### Vertical System

Vertical systems used in hydroponic farming are a contemporary approach to agriculture that involve growing crops in vertically stacked layers, often in an urban setting [198]. The concept has garnered immense interest from a diverse range of experts, including environmentalists, urban farmers, architects, agronomists, and public health professionals, who are keen to explore ways of addressing the challenges of food shortages in a rapidly urbanizing world [153]. Although the idea of vertical farming is not novel, it has recently gained renewed interest. In 1980, Åke Olsson, a Swedish ecological farmer, proposed vertical farming as a means to produce vegetables in cities [5,199]. In the early 2000s, Dickson Despommier, an American ecological professor, revitalized the concept with new vigor. He defined vertical farming as “the mass cultivation of plant and animal life for commercial purposes in skyscrapers” [199]. A vertical farm, utilizing advanced greenhouse technology, such as hydroponics and aeroponics, can, theoretically, produce fish, poultry, fruit, and vegetables. The concept of vertical farming has led to significant advancements in various fields, including robotics, aeroponics, aquaponics, and hydroponics. Several countries, such as Korea, Japan, China, Germany, the United Arab Emirates, China, France, India, Sweden, Singapore, and the United States, have convened to discuss and explore the potential of vertical farming [153,199].

According to the literature, three distinctive types of vertical farming models can be categorized [198,200]. The first type of vertical farming involves the construction of tall structures with multiple levels of growing beds, often equipped with artificial lights. This farming method has demonstrated high efficiency and productivity and maximized space utilization while minimizing dependence on traditional agricultural inputs such as soil and water. It is commonly utilized in modestly sized urban farms worldwide and has been implemented in both new and repurposed buildings, including warehouses [153,159]. The second type of vertical farming occurs on the rooftops of various commercial and residential structures, including restaurants, grocery stores, and both old and new buildings. This model offers a sustainable approach to producing fresh produce in urban areas, and it also helps to mitigate the urban heat island effect [198,199]. The third type is the visionary multistory building. This method involves constructing high-rise buildings that are specifically designed for agricultural purposes, featuring multiple floors of growing areas. Although still in its early stages, this model has the potential to revolutionize food production in urban areas by providing large quantities of fresh produce at scale [153,199].

### 4.2. Aeroponics

Aeroponics is an innovative and highly efficient method that involves the suspension of plant roots in the air, with a nutrient-rich water solution being misted onto the roots using advanced pumps, timers, and spray nozzles [201]. This process delivers highly oxygenated mists of water and nutrients to the roots of the plants at timed intervals, resulting in faster growth and increased yield. Aeroponics reduces water usage by up to 90% compared to conventional farming methods. This is because the water is recycled and reused in the system, and the misting process ensures that the plants receive just the right amount of water and nutrients they need to thrive [202]. Additionally, this method is used to grow a wide range of crops, including fruits, vegetables, and herbs. With the help of advanced technology, environmental conditions such as humidity, temperature, airflow, and light intensity can be precisely controlled using specific tools and systems. This means plants can be grown throughout the year, regardless of the external climatic conditions [203].

### 4.3. Aquaponics

Aquaponics is an innovative, sustainable agricultural technique that integrates hydroponics and recirculating aquaculture to form an integrated system capable of treating water before recycling it in a fish tank [204]. This reduces the negative environmental impacts associated with intensive fish and crop production and enhances crop yield [205,206]. This system relies on a biofilter and hydroponic section where plants absorb dissolved fish waste and products of microbial activity [207]. At the same time, certain volatile substances, such as CO_2_, CH_4_, N_2_, N_2_O, and NH_3_, are removed through gas volatilization [204]. The crucial components of an aquaponic system are the fish-rearing tank, the settler, the biofilter, and the hydroponic unit. The hydroponic unit plays a crucial role in maintaining water quality, which is essential for fish rearing, and is responsible for water loss through plant evapotranspiration. Therefore, special attention must be paid to the design and operation of the hydroponic system, as it directly influences the sustainability of the entire process in terms of water consumption and system management costs [204].

## 5. Performance of Microalgae in Hydroponic Systems

The World Health Organization advocates for the consumption of nutritionally rich vegetables with a daily intake of over 400 g per person [208]. Vegetables are rich in bioactive compounds called phytochemicals, such as phenolic acids, flavonoids, and carotenoids. These compounds are known to be chemoprotective agents with several antioxidant properties promoting human metabolism and overall well being [209]. In the last two decades, there has been a notable incline in the demand for high-quality vegetables attributed to the growing population’s interest in organoleptic, nutritional, and functional values [210]. However, variations in environmental conditions, such as water scarcity, temperature, humidity, and soil salinity, can stress crops, resulting in significant changes in the biochemical composition of fresh produce [156]. In this regard, numerous scientific studies have indicated that the hydroponic cultivation of vegetables results in a superior quality than those grown in conventional soil-based methods. Recent studies show that certain high-quality vegetables grown using hydroponic systems have significantly higher nutritional value owing to an increased concentration of bioactive compounds [34].

Furthermore, adding microalgae as a plant growth stimulator in hydroponic systems can promote growth and enhance the quality of vegetables. The potential application of cyanobacteria and microalgae in hydroponic systems can be accomplished either by mixing live algae cells in a nutrient solution or by applying cellular extracts and hydrolysates on foliage via foliar spraying [27]. Microalgal photosynthesis produces oxygen, preventing anaerobiosis in crops’ root systems and reducing sulfide injury in sulfate-reduction-prone plants [211]. Carbon (C) sources from crop root respiration and exudation can boost microalgal biomass and photosynthesis [162]. Moreover, microalgae can produce several organic nonmicrobial substances, such as phytohormones and protein hydrolysates, actively promoting plant growth [27]. However, microalgae contain a high lipid content with enriched unsaturated fatty acids and phycotoxins, which is uncommon in vegetables [212].

### 5.1. Incorporating Microalgae and Hydroponics in Circular Bioeconomy and Sustainability

The concept of a circular bioeconomy integrates organic waste management and food production, which can help to reduce the dependency on chemical fertilizers in agriculture [213]. Incorporating biorefinery and waste management principles, a circular bioeconomy model must also address social aspects, such as cascading, circular product design and product use [214]. Hydroponic systems play a crucial role in achieving a circular bioeconomy by circularly utilizing biomass or organic waste. Emerging technologies, such as microalgae-based fertilizers, are promising in addressing several concerns associated with the circular bioeconomy. Therefore, sustainable food systems that are part of a bioeconomy can enhance resilience and promote a more ecofriendly approach [215]. However, the application of this technology is still in its nascent stage and requires further exploration [216]. Furthermore, the mass cultivation of microalgae in the current system necessitates huge capital investments, particularly during the upstream growth and downstream harvesting stages [217].

A cocultivation system of microalgae in hydroponic systems produces higher biomasses and yields high-quality vegetables. Consequently, this cocultivation system offers a cost-effective, sustainable, and environmentally friendly approach [38]. Barone et al. [162] determined the potential of cocultivating microalgae *C. vulgaris* and *S. quadricauda* with tomato plants; the dry biomass yields for *C. vulgaris* and *S. quadricauda* were 0.77 ± 0.07 g L^−1^ and 1.02 ± 0.06 g L^−1^, respectively, and the corresponding volumetric productivity of the biomasses were 0.019 ± 0.004 g L^−1^ day^−1^ and 0.022 ± 0.002 g L^−1^ day^−1^, respectively (Table 5). Similarly, Mata et al. [218] reported that *C. vulgaris* ranged from 0.02 g L^−1^ day^−1^ to 0.20 g L^−1^ day^−1^ and *Scenedesmus* sp. from 0.03 g L^−1^ day^−1^ to 0.26 g L^−1^ day^−1^. Zhang et al. [38] found that *C. infusionum* in a simple ecohydroponic system with tomato produced 0.032 g L^−1^ day^−1^. In addition to single-strain cultivation, the cocultivation of a microalgae consortium (*Chlorella* sp., *Scenedesmus* sp., *Synechocystis* sp., and *Spirulina* sp.) with tomato plant showed a significant dry biomass yield of 0.149 ± 0.024 g/m^2^/d. Even after harvesting the microalgae cells, the culture media were recycled to support the plant growth for 24 days under nutrient-deficit conditions [39]. These findings suggest that the cocultivation system can revolutionize sustainable agriculture by enhancing high microalgae biomass production and producing superior-quality vegetables in hydroponic systems.

The growth rate of the microalgae consortium with tomato was calculated at 0.057 day^−1^ OD_680_, which was about 8% higher than that of monoculture with 0.053 day^−1^ OD_680_ [39]. Similarly, higher growth rates of microalgae *C. infusionum*, *Chlorella* sp., and *Scenedesmus* sp., during cocultivation with tomato plants [38,162], with lettuce [219] in a hydroponic system. The availability of stable soluble C in the nutrient medium owing to the plant root respiration and exudation increased the algae growth rate. However, the lower growth rate of microalgae observed in the low concentrations of inoculum could be attributed to the fact that the algal concentration may not have been sufficient to create a positive interaction among the phototrophs. Therefore, maintaining an appropriate microalgae inoculum is essential to achieve the requisite synergistic effects for the success of the higher growth benefits.

Agricultural wastewater from a commercial hydroponic greenhouse is one of the leading significant causes of environmental pollution due to the availability of unused nutrients, dissolved salts, and organic matter. Salazar et al. [220] estimated the bioremediation potential of microalgae *Tetradesmus obliquus* sp. NIVA-CHL107 on hydroponic wastewater, notably, the 100% removal efficiency of N and P was recorded. *T. obliquus* sp. NIVA-CHL107 produced a maximum yield of 6.2 g L^−1^ DW biomass. The composition of carbohydrates (21.4% and 64%), fatty acids (4.2% and 4%), proteins (52.8% and 17%), and carotenoids (6.3% and 1.8%) in the biomass varied distinctly at the exponential and stationary phase, respectively. Some studies often insist that microalgae can thrive and impact water quality parameters such as pH, dissolved oxygen (DO), nutrient consumption, and competition with target crops. Therefore, it is crucial to maintain optimal levels of microalgae population in hydroponic water systems. However, microalgae can act as a buffer, making chemical sanitization more cost-effective than bleach treatment [221]. The elimination rate of total dissolved solids, total N, and total P is significantly improved by *C. vulgaris*, especially with total N and P removal efficiency at 97.6% and 98.8%, respectively [211]. Similarly, *C. vulgaris* has shown N and P removal efficiency rates ranging from 92.41% to 97.48% and 96.41% to 99.96%, respectively, while promoting the high number of leaves (18.56%), total fresh weight (17.13%), and root volume (36.98%) in Swiss chard [222].

**Table 5:** Influence of the cocultivation of microalgae on vegetable growth parameters, nutrient removal efficiency, and microalgae biomass accumulation in the hydroponic system. TN: total nitrogen; TP: total phosphorus; TDS: total dissolved solids.

Microalgae	Plants	N Removal	P Removal	Leaf Number	Fresh Weight	Dry Weight	Shoot Length	Root Length	Biomass Productivity	Biomass Yield	Other Results	Reference
*Chlorella vulgaris*	Swiss chard	TN: 92.41–97.48%	TP: 96.41–99.96%	18.56%	17.13%	-		36.98%	-	-		[222]
*C. vulgaris, Scenedesmus quadricauda*	Tomato	-	-	-	11.95 g	0.90 g		130%	0.77–1.02 g L^−1^	0.019–022 g L^−1^ day^−1^		[162]
*Chlorella infusionum*	Tomato	TN: 84%	TP: 44%	-	-	-		22.95 g	-	32–54.24 g dm^−3^ d^−1^		[38]
*Chlorella* sp., *Scenedesmus* sp., *Synechocystis* sp., *Spirulina* sp.	Tomato	NO_3_: 41–84%, NH_4_: 88–99%	PO_4_^3^⁻: 60–94%	31–43%	2.19–6.05 g	0.16–0.50 g	17.37–19.25 cm	10.37–25.75 cm	1.12–3.18 g	0.066–0.149 g day^−1^	K removal: 82–95%,	[39]
*C. vulgaris*	Lettuce	-	-	17.75–20.25 plant^−1^	237.56–243.31 g plant^−1^	6.53–7.29 g plant^−1^	-	-	-	-	-	[223]
*C. vulgaris* (UTEX 2714)	Arugula, Purple kohlrabi, Lettuce	TN: 94.6–97.6%	TP: 92.9%	-	-	-	0.43–0.80 cm·d^−1^	0.43–1.85 cm·d^−1^	0.40–0.71 g·L^−1^	0.78–1.86 g·m^−2^·d^−1^	Dissolved Oxygen: 7.89–8.23 g·mL^−1^, TDS removal: 56.7%	

### 5.2. Plant Growth Promotion

As previously discussed, algae biostimulants are particularly effective in hydroponic cultures, where they have been shown to enhance crop performance and reduce the use of fertilizer and nutrient solution concentration. Specifically, they have been observed to positively impact root and shoot growth, enhancing water uptake and increasing tolerance to abiotic stress [27].

#### 5.2.1. Productivity

Plants that are exposed to varying concentrations of microalgae consortium ranging from 0.2 to 0.8 mg ml^−1^ significantly increased the tomato crop productivity by 437%, yielding up to 0.328 ± 0.087 g/m^2^/d, compared to the uninoculated control, which only produced 0.061 ± 0.003 g/m^2^/d in HPS (hydroponic system) [39]. Similarly, *C. vulgaris* was found to enhance lettuce and purple kohlrabi productivity by 33% (0.95 ± 0.27 kg m^−2^) and 11% (2.16 ± 0.46 kg m^−2^), respectively, compared to the microalgae-free control, which only yielded 0.71 ± 0.21 kg m^−2^ and 1.94 ± 0.42 kg m^−2^, respectively. Furthermore, Ergun et al. [223] reported that *C. vulgaris* also increased lettuce yield from 238 to 243 g plant^−1^, i.e., 9 to 16% higher than the control, ranging from 206 to 223 g plant^−1^, respectively. These findings suggest that the increase in plant productivity was mainly attributed to the oxygen produced by microalgae during photosynthesis and the CO_2_ fertilization from crop root respiration. The coutilization of nutrients by microalgae and plants in the HPS leads to high efficiency in N and P utilization. Therefore, the symbiotic relationship between microalgae and crops promotes plant productivity and reduces nutrient load, thus supporting sustainable practices in urban agriculture.

#### 5.2.2. Biomass

Microalgae colonization in the plant root system can facilitate root respiration, increasing energy availability for complex metabolic functions. This has resulted in faster uptake and nutrient transmission (N, P, K), leading to the development of plant biomass [36]. Supraja et al. [39] reported increased fresh weight of plant shoots (6.05 ± 0.42 g plant^−1^) and roots (3.82 ± 0.44 g plant^−1^), as well as the dry weight of plant shoots (0.502 ± 0.145 g plant^−1^) and root (0.105 ± 0.022 g plant^−1^), leading to higher plant productivity (0.328 ± 0.087 g/m^2^/d) in tomatoes leading through cocultivation with microalgae consortiums. Furthermore, the rapid assimilation of nutrient uptake often promotes the accumulation of nutrients inside vacuoles, leading to a higher fresh weight of roots than shoots [94]. Similar results were found by researchers who demonstrated the positive effect of *Chlorella* sp. and *Scenedesmus* sp., over the tomato plant fresh and dry matter [38,180]. Moreover, Zhang et al. [38] reported that using *C. infusionum* in an ecofriendly hydroponic cocultivation system has also resulted in highly developed roots with more dry weight and greater crop productivity for tomato plants.

#### 5.2.3. Plant Height

Assessing plant height is a complex process involving various factors, including shoot and root length. Huo et al. [211] revealed that the microalgae consortium improved the shoot growth rate of purple kohlrabi (0.80 cm d^−1^) compared to the control (0.70 cm d^−1^) in hydroponic cultivation. Similarly, Barone et al. [162] reported an 130% increase in the total plant length of tomatoes cocultivated with *C. vulgaris* and *S. quadricauda*, and Cortés-Jiménez et al. [224] reported an increase in the shoot length of tomato seedlings inoculated with *C. vulgaris* in the hydroponic system. These significant results suggested that the association of microalgae with plants can increase plant root respiration and nutrient uptake, resulting in better nutrient assimilation and improved plant height. Additionally, this putative mechanism has been attributed to the secretion of secondary metabolites and allelochemicals, such as phytohormones, which also enhance plant growth [36]. In particular, tomatoes grown with a microalgae consortium exhibited a shoot length of 19.25 ± 1.14 cm plant^−1^ and root length of 25.75 ± 2.3 cm plant^−1^, representing an increase of 52.4% and 217% compared to the microalgae-free treatment, respectively [39]. These findings underscore the potential of the microalgae consortium as a growth-promoting agent for plants under hydroponic cultivations.

#### 5.2.4. Leaf Count

The assessment of plant growth often involves an increase in the number of leaves [224]. In one study, a microalgal consortium at a concentration of 0.8 mg mL^−1^ increased the number of leaves per plant to 43.5 ± 4.43, which was 65.7% greater than the control (i.e., without microalgal consortium). Escalante et al. [225] reported a similar increase in the number and length of leaves in tomato plants grown in a hydroponic system inoculated with *C. vulgaris*. In addition, Bharti et al. [36] reported a positive interaction between a microalgal consortium and the plant, resulting in healthier leaves with higher chlorophyll contents. This effect was attributed to the availability of C- and N-based metabolites in the plant root periphery colonized by microalgae, which increases the leaf chlorophyll contents [51].

#### 5.2.5. Pigmentation

Chloroplasts are organelles in plant cells that contain pigments such as chlorophyll and carotenoids, which absorb light energy to facilitate photochemical redox reactions during photosynthesis [226]. Various factors, including the quality of light, the uptake of essential nutrients such as N and P, and the nature of the photosynthetic organisms, influence the pigment concentration in phototrophs [226]. The positive interaction between the algal consortium and plants in a hydroponic cocultivation system facilitates the efficient uptake of essential nutrients, which are then incorporated into the backbone of the pigments formed [227]. Additionally, the availability of C- and N-containing metabolites provided by the microalgae increases plant biomass and chlorophyll content [228]. Supraja et al. [39] showed that a hydroponic cocultivation system of microbial consortium and tomato plants could significantly increase the concentration of Chl a and Chl b pigments by up to 1.55 ± 0.031 mg g^−1^ and 0.39 ± 0.033 mg g^−1^, respectively, and carotenoids were observed at a concentration of 0.013 ± 0.0003 mg g^−1^. Additionally, algal biomass in the same cocultivation unit also showed an increase in Chl a concentration of up to 0.51 ± 0.010 mg g^−1^, with Chl b and carotenoids at 0.27 ± 0.062 mg g^−1^ and 0.006 ± 0.0002 mg g^−1^, respectively. A hydroponic cocultivation system employing *Chlorella* sp. and *Scenedesmus* sp., has been shown to improve the health of tomato plants in terms of chlorophyll content, confirming the beneficial effects of cocultivating both plants and microalgae. These findings could have significant implications for the agricultural industry, as hydroponic cocultivation systems could be potentially used to improve crop yields and sustainability.

### 5.3. Nutrient Reduction

In recent times, there has been a growing interest in the use of microalgae extracts as biostimulants to enhance agricultural yields and reduce the need for chemical fertilizers because of their favorable impact on plant growth and their ability to induce tolerance toward environmental stressors [229]. Proper nutrient management in a hydroponic system (from 100 to 200 mg L^−1^) induces lettuce growth and development, higher fresh weights (from 12 to 41.9 kg m^−2^), and increased numbers of leaves (19.1 to 22.5) [230,231], and vice versa, higher mineral salts coinduced by excess sodium chloride, which jointly trigger a low water potential and ion-specific imbalance, induce morpho-physiological and metabolic changes that lead to plant growth inhibition [232,233,234]. Despite this, N remains essential in chlorophyll formation. Studies have shown that decreased N levels significantly reduce photosynthesis and inhibit crop yield [231]. Numerous research studies have established the efficacy of microalgae-based biofertilizers and biostimulant properties in fostering the growth of vegetable crops and minimizing the use of mineral nutrients. For instance, *C. vulgaris* incorporation reduced 60% of fertilizer and produced better quality leaves by increasing total soluble solids (brix) and vitamin C contents in hydroponically grown lettuce [223]. *Chlorella* sp. and *Anabaena* sp. extracts promoted lateral and fibrous root growth while inhibiting root elongation and increasing cucumber seedlings’ length by 81.7% and 58.3%, respectively [235].

### 5.4. Dissolved Oxygen Content

Microalgae exhibit a greater photosynthetic efficiency than higher plants. Their exceptional photosynthetic efficiency can be attributed to their capability of utilizing a diverse range of antenna pigments that capture a more significant amount of light energy, and several carbon dioxide (CO_2_)-concentrating mechanisms facilitate CO_2_ fixation around Rubisco to generate molecular oxygen [236]. Notably, microalgae produce oxygen (O_2_) at high rates (up to 10 mg L^−1^ min) by fixing 1 mol of CO_2_ to generate 1 mol of O_2_ through photosynthesis [237]. This attribute is vital in hydroponics, whereby the oxygen produced by microalgae helps prevent anaerobiosis in the root system of crops [38]. Generally, artificial aeration systems are widely used to maintain dissolved oxygen levels (4 and 6 mg L^−1^) in the hydroponic nutrient solution for the entire plant growth cycle. A study found that coculture of a microalgae consortium of *Chlorella* sp., *Scenedesmus* sp., *Synechocystis* sp., and *Spirulina* sp. with tomato enhanced DO levels ranging from 9.5 to 12 mg L^−1^ at the concentrations of 0.2–0.8 mg mL^−1^ of microalgae inoculum in the hydroponic nutrient solution. In contrast, the control group had a maximum DO level of 7.5 mg L^−1^ on the 36th day after plant transplantation [39]. The root respiration rate of CO_2_ was 23 ± 6 ng kg^−1^ s^−1^ in the absence of aeration, which is a lower value than the corresponding rates of 92 ± 29 ng kg^−1^ s^−1^ in the presence of aeration and 81 ± 36 ng kg^−1^ s^−1^ in the microalgae coculture medium, because the crop root respiration rate is directly proportional to the level of DO in the nutrient solution. As a result, there is high CO_2_ production due to the high respiration rate of the developed root system in nutrient solutions with high DO contents. These results indicate that cocultivation of microalgae with plants in the hydroponic nutrient solution is crucial for improving DO levels, root respiration rates, and growth, leading to high crop yields and productivity [38,238].

## 6. Concluding Remarks

Achieving food security is a complex issue that requires a holistic approach. Technological advancements in food production systems can be crucial in enabling food-insecure populations to attain food self-sufficiency, especially regarding nutrition security. Food production objectives prioritize output optimization across different production systems rather than maximizing productivity. Modern agriculture approaches, such as hydroponics technology, present a promising solution to ensuring food security and sustainability, especially in highly populated cities. In recent years, there has been a growing trend toward using microalgae inoculants as substitutes for chemical fertilizers and nonrenewable resources. Microalgae and cyanobacteria possess the potential ability to enhance plant growth and provide systemic immune resistance against a range of various environmental stresses. However, the commercial success of microalgae-based agriculture products depends on the cost efficiency and low energy footprints in biomass production processes. Therefore, it is necessary to integrate bioremediation and biorefinery models to improve the commercial feasibility of microalgae-based agro-products. Closed-loop hydroponic systems that cocultivate microalgae and cyanobacteria might effectively deliver significant economic advantages by yielding multiple products, such as nutrient-rich crops and microalgae, thereby contributing to global food security. Additionally, they can help reduce the expenses associated with the upstream cultivation process and the downstream steps of wastewater treatment by decreasing the nutrient load.

## Figures and Tables

**Table 1:** Application of microalgae and cyanobacteria as biostimulants and their impact on plant growth parameters under laboratory or greenhouse conditions. Improved growth parameters are indicated by “+”.

Microalgae	Plants	Outcomes					Reference
		Germination	Shoot/Root Length	Plant Biomass	Nutrient Content	Other Results	
**Live cell suspensions or fresh biomass**							
*Anabaena laxa*, *Calothrix elenkinii*	*Coriandrum sativum*, *Cuminum cyminum*, *Foeniculum vulgare*	+	+	+		Increased peroxidase activity in shoots and roots and antifungal activities against *Macrophomina phaseolina* and *Fusarium moniliforme*	[35]
*Anabaena torulosa*, *Trichormus doliolum, A. laxa*	*Chrysanthemum morifolium*		+	+		Enhanced leaf pigments, IAA production, and PEP carboxylase activity	[36]
*Chlorella fusca*	*Spinacia oleracea*			+	+	Increased plant yield, leaf width, thickness and number, and resistance to gray mold disease	[37]
*Chlorella infusionum*	*Solanum lycopersicum*		+	+	+		[38]
*Chlorella* sp., *Scenedesmus* sp., *Synechocystis* sp., *Spirulina* sp.	*S. lycopersicum*	+	+	+	+	Enhanced chlorophyll pigments and dissolved oxygen	[39]
*Chlorella vulgaris*	*Hibiscus esculentus*	+	+			Increased number of flower buds	[40]
*C. vulgaris*	*Triticum aestivum* L.			+	+	Increased plant growth, leaf area, and root hair production	[41]
*Microcystis aeruginosa*, *Anabaena* sp., *Chlorella* sp.	*Zea mays*	+	+			Inhibited the growth of pathogenic bacteria and fungi	[42]
**Dry biomass, cell extracts, or hydrolysates**							
*Tetradesmus dimorphus*	*S. lycopersicum*	+	+	+	+	Increased number of flowers and branches	[22]
*C. vulgaris*	*Lactuca sativa* L.	+	+	+	+	Increased leaf chlorophyll, carotenoid, and protein content	[43]
*C. vulgaris*, *Limnospira platensis*	*Z. mays* L.	+				Enhanced early seedling growth and improved yield characteristics	[44]
*Chlorococcum* sp., *Micractinium* sp., *Scenedesmus* sp., *Chlorella* sp.	*S. oleracea* L.	+		+	+	Synthesis of cytokinins (trans-zeatin, DHZR, tZMP, iP, iPA, and iPAMP), gibberellins (GA1, GA3, GA4, GA20, and GA29), auxin (IAA), and abscisic acid (ABA)	[45]
*Nannochloropsis oculata*	*S. lycopersicum* cv. Maxifort		+	+	+	Improved the fruit quality through an increase in sugar and carotenoid contents	[46]
*Nostoc commune*	*Oryza sativa* cv. Shiroodi L.	+	+	+			[47]
*L. platensis*	*Raphanus sativus*	+	+	+		Enhanced leaf pigments	[48]
*L. platensis*	*Vigna mungo* L.	+	+	+	+		[49]
*Ulothrix* sp., *Pinnularia* sp., and *Oscillatoria* sp.	*S. lycopersicum*, *Capsicum annuum*, *Solanum melongena*	+	+	+		Improved disease resistance	[50]

**Table 2:** Phytohormones identified in microalgae and cyanobacteria.

Species	Metabolites	Targets Promoted	Reference
**Auxin**			
*Auxenochlorella pyrenoidosa, Scenedesmus quadricauda*	Indole-3-acetic acid (IAA), indole-3-butyric acid (IBA)	Lipid content and production	[59]
*C. fusca*, *C. vulgaris*, *Scenedesmus obliquus*, *Synechococcus nidulans*, *Spirulina* sp. LEB 18	IAA	Carbohydrate, protein	[60]
*C. vulgaris*	IAA, IBA, phenylacetic acid (PAA)	Cell divisions, proteins, chlorophylls, monosaccharides	[61]
*Desmodesmus* sp.	IAA, IBA, IPA	Biomass, lipids, fatty acids	[62]
*Dunaliella salina*	IAA	Growth, β-carotene	[63]
*C. vulgaris*	IAA	Biomass, lipid content and productivity	[64]
*Nannochloropsis oceanica*	IAA	Growth, lipid	[65]
*N. oculata*	IAA	Cell division, chlorophyll-a	[66]
*S. obliquusi*, *Pilidiocystis multispora*, *C. vulgaris*	IAA	Growth, PUFAs	[67]
*S. obliquus*	IAA	Growth, fatty acid, protein, carbohydrate content	[68]
*S. quadricauda*	Auxins	Cell divisions, growth, biomass, chlorophyll, carotenoids, fatty acids	[69]
*Scenedesmus* sp., *Chlorella sorokiniana*	IBA, NAA	Lipid	[70]
**Cytokinin**			
*Tetradesmus obliquus*	Kinetin, zeatin	Biomass, lipid, carbohydrate	[71]
*C. fusca*, *C. vulgaris*, *S. obliquus*, *S. nidulans*, *Spirulina* sp. LEB 18	Trans-zeatin	Carbohydrate, protein	[60]
*Auxenochlorella protothecoides*	Cytokinin	Biomass, lipid	[72]
*C. vulgaris*	Zeatin	Cell divisions, carotenoids	[73]
*C. vulgaris*	Benzyladenine, trans-zeatin, 2-methylthio-trans-zeatin	α-Linolenic, linoleic, palmitic, oleic, and stearic acids	[74]
*Desmodesmus* sp.	6-benzylaminopurine, Thidiazuron	Biomass, lipids, fatty acids	[62]
*D. salina*	Kinetin	Growth, β-carotene	[63]
*Nostoc muscorum*	Kinetin	Biomass, carotenoids	[75]
**Gibberellic acid**			
*Chlorella ellipsoidea*	Gibberellic acid (GA)	Growth, lipid	[76]
*A. pyrenoidosa*	GA3	Growth, lipid	[77]
*C. vulgaris*	GA	Cell divisions, carotenoid	[73]
*Isochrysis galbana*	GA3	Biomass, chlorophyll a, protein, lipid, PUFAs	[78]
*Monodopsis subterranea*	GA	Biomass, total fatty acid, eicosapentaenoic acid	[79]
*N. oculata*	GA	Cell diameter, lipid	[66]
**Ethylene**			
*C. vulgaris*	Ethephon	SFAs, a-tocopherol, c-aminobutyric acid, asparagine, proline	[80]
*Haematococcus lacustris*	1-Aminocyclopropane-1-carboxylic acid (ACC)	Astaxanthin	[81]
*H. lacustris*	Ethylene	Astaxanthin, lipid	[82]
*Monoraphidium* sp.	Ethylene	Lipid	[83]
**Abscisic acid**			
*A. pyrenoidosa*	Abscisic acid (ABA)	Lipid	[84]
*C. vulgaris*	ABA	Biomass, total fatty acid	[85]
*C. vulgaris*	ABA	Fatty acids	[74]
*Chromochloris zofingiensis*	ABA	Growth, fatty acid, pigmentation	[86]
*D. salina*	ABA	Growth, β-carotene	[63]
**Salicylic acid**			
*Chlorella* sp.	Salicylic acid (SA)	Cell growth	[87]
*C. zofingiensis*	SA	Cell growth, total fatty acids, astaxanthin	[88]
*H. lacustris*	SA	Biomass, astaxanthin	[89]
**Jasmonic acid**			
*C. vulgaris*	Jasmonic acid (JA)	Cell divisions, carotenoid	[73]
*H. lacustris*	Methyl jasmonate (MJ)	β-Carotene, lutein	[89]
*M. subterranea*	MJ	Biomass, total fatty acid, eicosapentaenoic acid	[79]
*Stauroneis* sp.	MJ	Lipids and pigments	[90]

**Table 3:** Effect of microalgae biostimulants on the mitigation of abiotic stress in various crop plants [32].

Microalgae	Plants	Stress	Tolerance
*Dunaliella salina, Phaeodactylum tricornutum*	Bell pepper	Salinity	Reduced production of superoxide radicals, decreased lipid peroxidation, and increased antioxidant enzyme activity.
*D. salina*	Wheat	Salinity	Improved seed germination and coleoptile height. Enhanced the accumulation of proline and ROS antioxidant enzymes like catalase (CAT), peroxidase (POD), and superoxide dismutase (SOD).
*Nannochloris* sp.	Tomato	Water stress	Enhanced root length, leaf number, and leaf area.
*Chlorella vulgaris*	Guar	Drought	Increased shoot length, fresh and dry weights of shoot and root. Stimulated the accumulation of relative water content, total phenolic content, and ROS scavengers, such as SOD, CAT, ascorbate peroxidase (APX), and glutathione reductase (GR).
*C. vulgaris*	Onion	Drought	Increased growth parameters, nutrients, and accumulation of carbohydrates.
*C. vulgaris*	Guar	Salinity	Increased photosynthetic pigments and induced antioxidant enzymes, such as SOD, CAT, GR, and APX, and decreased MDA, NA^+^, and Ca^−^ ions.

**Table 4:** Various crop species are successfully grown using a hydroponic system.

Crops	Crop Names	References
Cereals	*O. sativa*, *Z. mays*	[163,164]
Condiments/herbs	*Coriandrum sativum*, *Trigonella foenum-graecum*, *Petroselinum crispum*, *Mentha piperita*, *Rosmarinus officinalis*, *Ocimum basilicum*, *Origanum vulgare*	[165,166,167,168,169,170]
Flower/ornamental crops	*Tagetes* sp., *Rosa* sp., *Dianthus* sp., *Chrysanthemum* sp.	[36,171,172]
Fodder crops	*Sorghum bicolor*, *Medicago sativa*, *Cynodon dactylon*, *Axonopus* sp.	[173,174,175]
Fruits	*Fragaria ananassa*	[176]
Leafy vegetables	*L. sativa*, *S. oleracea*, *Apium graveolens*, *Atriplex* sp.	[177,178,179,180]
Medicinal crops	*Aloe perfoliata*, *Coleus* sp.	[181,182]
Microgreens	*R. sativus*, *Brassica oleracea*, *Lepidium sativum*, *Eruca sativa*, *Daucus carota*, *Helianthus annuus*, *Amaranthus* sp., *Fagopyrum esculentum*, *Ocimum basilicum*, *Rumex acetosa*, *T. aestivum*, *Medicago sativa*, *Brassica* sp., *Trifolium* sp.	[3,152,169,183,184,185,186,187,188,189,190,191,192]
Vegetables	*S. lycopersicum*, *Capsicum* sp., *S. melongena* L., *Phaseolus vulgaris*, *Beta vulgaris*, *Cucumis* sp., *Allium fistulosum* L.	[39,193,194,195,196,197]

## Data Availability

All data required to evaluate the conclusions of this paper are included in the main text.
